# Mapping Antimicrobial Synergism in Sorbate-Based Ternary Preservative Systems Against *Listeria innocua* and *Salmonella* Typhimurium Using Multivariate Analysis

**DOI:** 10.3390/foods15162793

**Published:** 2026-08-10

**Authors:** Ricardo H. Hernández-Figueroa, Elizabeth Baltazar-Fernández, Aurelio López-Malo, Aarón Romo-Hernández, Emma Mani-López

**Affiliations:** Departamento de Ingeniería Química, Alimentos y Ambiental. Universidad de las Américas Puebla, Ex Hacienda Sta. Catarina Mártir, San Andrés Cholula 72820, Puebla, Mexicoaaron.romo@udlap.mx (A.R.-H.); emma.mani@udlap.mx (E.M.-L.)

**Keywords:** ternary antimicrobial mixtures, synergism, Partial Least Squares (PLS), foodborne pathogens, potassium sorbate, natural preservation

## Abstract

Designing multi-ingredient preservation systems is crucial for reducing the use of synthetic additives while maintaining food safety. This study evaluated the antimicrobial efficacy of ternary mixtures combining two natural antimicrobials (thymol, carvacrol, eugenol, citral, vanillin) and potassium sorbate (PS) against *Listeria innocua* and *Salmonella* Typhimurium at pH 4.5 and 5.5. The fractional inhibitory concentration index (FICI) and the total minimum inhibitory concentration (MIC) were determined to identify optimal synergistic combinations. Multivariate analysis, including Principal Component Analysis and Partial Least Squares (PLS) regression with standardized coefficients, was applied to decipher the relative impact and hierarchy of the predictor variables. At pH 4.5, concentrations of PS ≤ 64 ppm combined with any of the natural components resulted in higher synergistic mixtures (low FICI 0.318–0.378) for *L. innocua*. For *Salmonella*, only combinations of thymol, carvacrol, eugenol, and PS ≤ 32 ppm obtained the lowest FICI (0.252–0.344) at pH 4.5, while at pH 5.5, 128 ppm PS, thymol, and vanillin were required for a similar FICI (0.363). The PLS models revealed a distinct shift in variable importance between the synergistic index and the MIC. For the FICI model, mixture components exerted the primary influence; potassium sorbate displayed the highest predictive weight (standardized coefficient: +0.8736), followed by eugenol (+0.7757), carvacrol (+0.6206), and citral (+0.5119). This indicates that maximum synergism (lowest FICI) is constrained to lower fractional concentrations of natural compounds, thereby avoiding saturation of the cellular target site. Bacterial species (+0.2418) and pH (+0.3146) contributed less, indicating a homogeneous effect across the tested bacteria. For the total MIC model, potassium sorbate (+0.4701) and vanillin (+0.4492) regulated the antimicrobial quantity requirements. For the strains evaluated, the coefficients of the bacterial type in both models indicate that the ternary mixtures performed similarly. These findings demonstrate that integrating multivariate PLS modeling provides a robust framework for optimizing natural-synthetic antimicrobial blends, significantly reducing dependence on potassium sorbate through tailored synergism.

## 1. Introduction

Foodborne pathogens continue to represent a major challenge for the food industry and public health systems worldwide [1]. Among them, *Listeria monocytogenes* and *Salmonella* spp. are responsible for a significant proportion of foodborne outbreaks and recalls due to their ability to survive under diverse environmental conditions and persist throughout food processing and storage. Recently, in 2026, *Salmonella* Typhimurium and *Salmonella* Newport infections were linked to moringa leaf powder and *Moringa oleifera* capsules, which resulted in product recalls in the United States [2,3], while in Germany, a similar outbreak was associated with *Salmonella* Bochum in chocolate products [4]. *Listeria* outbreaks have also recently been reported and have resulted in product recalls. In May and June 2026, requesón (cottage cheese/soft ricotta cheese) and other cheese products (soft and semi-soft “Spanish style cheese varieties”, mild cheese varieties, hard cheese varieties, smoked Cheddar cheese varieties, flavored cheeses, pepper cheese varieties, and smoked pepper cheese varieties) were recalled from the market following concerns about *Listeria* contamination [5,6]. In June 2024, inside the European Union, a prolonged multi-country outbreak of *Listeria monocytogenes* ST173 was linked to the consumption of fish products [7]. Developing preservation strategies to control foodborne pathogens while meeting consumer demand for safer and more natural foods remains a priority for the food industry [8,9,10].

Consumer preference for clean-label products drives the search for preservation strategies that reduce synthetic preservatives. Essential oils are promising broad-spectrum antimicrobials due to their complex composition, including alcohols, aldehydes, phenols, acids, esters, and ketones [8]. Their antimicrobial activity is related to primary constituents such as thymol, carvacrol, citral, eugenol, and vanillin, which target foodborne pathogens and spoilage microorganisms [9,10]. Research on plant-derived natural antimicrobials has gained increasing attention due to growing consumer demand for clean-label foods. Combinations of volatile monoterpenes have been shown to exert potent, multi-target antimicrobial effects. Binary mixtures of thyme and oregano essential oils have shown significant synergistic activity against foodborne pathogens [11]. Addition of natural monoterpenes in chitosan films boosts combined bactericidal effectiveness [12]. Unfortunately, the concentrations of natural volatile compounds required to achieve effective antimicrobial activity often cause undesirable changes in the organoleptic properties of foods. Therefore, strategies are needed to incorporate these bioactive compounds at concentrations that ensure food safety while preserving the sensory quality and consumer acceptability of the products. Exploring the interactions between natural bioactive compounds and subinhibitory concentrations of chemical preservatives, such as potassium sorbate, represents a promising strategy for enhancing food safety while minimizing their impact on sensory quality. [13,14]. This approach may reduce the concentration of natural antimicrobials required for effective microbial control, thereby minimizing their impact on sensory quality while maintaining food safety and consumer acceptance. Therefore, current preservation strategies often combine natural antimicrobials with conventional preservatives to achieve synergistic effects, thereby reducing the concentrations required for effective microbial control. This approach is consistent with the principles of hurdle technology and can enhance microbial safety while minimizing undesirable sensory changes [7,11].

Essential oils exert antibacterial activity primarily through interactions with microbial cell membranes [15], and their constituents disrupt cytoplasmic membrane integrity, increase membrane permeability, and interfere with important metabolic processes [8]. Due to their hydrophobic nature, these constituents partition into the lipid bilayer, where they destabilize membrane structure and compromise selective transport mechanisms. These effects lead to leakage of intracellular components, loss of ions and macromolecules, disruption of energy metabolism, and ultimately bacterial cell death [15,16,17]. Gram-positive bacteria are generally more susceptible to these effects than Gram-negative bacteria, likely due to the absence of an outer membrane containing lipopolysaccharides, which can limit compound penetration [18]. Recent studies show that combining essential oil compounds with agents such as zinc, phenolic acids, or organic acids enhances antimicrobial efficacy [19,20,21]. Loading thymol or carvacrol into zinc-modified clays preserves their antimicrobial activity [19], while combinations with gallic or other organic acids achieve synergistic pathogen inactivation by disrupting membranes [20,21]. These findings support the use of ternary, multi-component systems to maximize antimicrobial performance.

Sorbates are widely used food preservatives and are generally recognized as safe by the U.S. Food and Drug Administration [22]. Among them, potassium sorbate is a commonly used conventional preservative in the food industry due to its antimicrobial activity, low cost, and regulatory acceptance [23]. Environmental factors, particularly pH, strongly influence the antimicrobial activity of sorbates, as their efficacy depends on the dissociation equilibrium of sorbic acid in aqueous media. In acidic conditions, the undissociated form predominates and exhibits higher antimicrobial activity. This pH-dependent behavior provides a rationale for combining potassium sorbate with natural antimicrobials to enhance antimicrobial performance while potentially reducing synthetic preservative concentrations [24]. Consistent with this synergistic concept, natural preservatives have also been reported to exhibit enhanced antimicrobial efficacy when used in combination rather than individually, although their application requires careful safety evaluation [25].

Several studies have demonstrated that combining potassium sorbate with natural antimicrobials enhances antibacterial activity compared with individual compounds. Improved activity against *Staphylococcus aureus* has been reported for combinations with aqueous and ethyl acetate fractions of *Stryphnodendron adstringens* [24]. Synergistic interactions (Fractional Inhibitory Concentration Index, FICI < 0.5) between cinnamaldehyde and potassium sorbate have been observed against *Salmonella* Typhimurium and *Staphylococcus aureus* in vitro and in food systems, including apple jam [26]. In addition, carvacrol combined with potassium sorbate has shown additive effects, contributing to the elimination of *Salmonella* Typhimurium in tomato paste under acidic conditions [27]. Collectively, these findings support the potential of potassium sorbate-based combinations with natural antimicrobials to enhance microbial control in food systems.

Although numerous studies have investigated binary combinations involving potassium sorbate or essential oil constituents, studies addressing ternary systems combining potassium sorbate with two natural antimicrobial compounds remain limited. In addition, the combined influence of environmental pH and target microorganism on the performance of such ternary systems has not been comprehensively evaluated [25,28,29].

Antimicrobial interactions are commonly evaluated using the FICI, which classifies effects as synergistic, additive, or antagonistic. However, when multiple compounds, target microorganisms, and environmental conditions are considered simultaneously, interpretation based solely on pairwise interaction indices may become limited. The susceptibility of *Salmonella* Enteritidis to combinations of potassium sorbate, vanillin, and citral was evaluated in culture media using the spiral gradient method and the Berenbaum design [30,31]. The Berenbaum design provides a robust mathematical framework for simultaneously assessing synergistic interactions among multiple antimicrobial agents through response surface analysis. The spiral gradient method is an efficient automated technique that generates a continuous exponential concentration gradient on a single agar plate, eliminating the discrete dilution steps required by conventional methods. The combination of these approaches revealed synergistic interactions that reduced the concentration required to inhibit bacterial growth in fruit purées.

Nevertheless, the increasing complexity of experimental designs involving multiple antimicrobials, microorganisms, and environmental factors generates multidimensional datasets that may not be fully captured by conventional interaction indices alone. In this context, multivariate tools such as Principal Component Analysis (PCA) and Partial Least Squares (PLS) regression offer complementary approaches for identifying patterns in antimicrobial behavior, classifying formulations, and determining the variables most strongly associated with antimicrobial performance. Advanced multivariate methods like Two-way Orthogonal Partial Least Squares Discriminant Analysis have successfully analyzed essential oils’ complex chemical profiles and identified key volatile combinations for synergistic effects against foodborne pathogens [32]. While these tools are necessary for natural extracts, their use in optimizing synthetic-natural ternary antimicrobial systems for food preservation is an innovative way to enhance predictive capabilities. These methods have been widely applied in food chemistry and process optimization, but remain underutilized for analyzing antimicrobial interaction systems in food preservation [33,34]. The objective of this study was to evaluate the antimicrobial effectiveness of ternary mixtures comprising potassium sorbate and natural antimicrobial compounds (thymol, carvacrol, citral, eugenol, and vanillin) against *Listeria innocua* and *Salmonella* Typhimurium at pH 4.5 and 5.5. Fractional inhibitory concentration indices (FICI) and minimum inhibitory concentrations were determined for the different formulations. In addition, principal component analysis and partial least squares analysis were used to identify interaction patterns, the main components associated with antimicrobial synergism, and the relationships between the composition of the antimicrobial mixture, environmental conditions, and inhibitory efficacy.

## 2. Materials and Methods

### 2.1. Microorganisms and Preparation of the Inoculum

Strains of *Salmonella* Typhimurium ATCC 14028 and *Listeria innocua* (ATCC 51742) were provided from the Laboratory of Food Microbiology (UDLAP) collection. The strains were grown on tryptic soy agar (TSA) slants (Merck, Darmstadt, Germany) at 35 °C for 24 h and cultivated continuously. The inoculum was prepared by transferring each bacterium into tryptic soy broth (TSB, Merck, Darmstadt, Germany) and incubating at 35 °C for 24 h to achieve a population of approximately 10^8^ CFU/mL in the stationary phase. *L. innocua* was selected as a surrogate microorganism because of its close genetic and physiological relationship with *Listeria monocytogenes*, making it a widely accepted model for evaluating antimicrobial activity [35].

### 2.2. Experimental Design

A chessboard design [36] was used to evaluate, at a water activity of 0.99, the effects of ternary mixtures at pH 4.5 and 5.5 on the inhibition of each microorganism under investigation. The ternary mixtures were prepared using potassium sorbate (Sigma Chemical Co., St. Louis, MO, USA) and two other natural antimicrobial agents. The natural antimicrobials evaluated were thymol, vanillin, eugenol (Sigma Chemical Co., St. Louis, MO, USA), carvacrol, and citral (Fluka Chemie AG, Buchs, Switzerland). The ternary combinations evaluated are presented in Table 1.

The concentrations to assess in the ternary mixtures were determined based on the minimum inhibitory concentrations (MICs) reported by Santiesteban et al. [37] for each antimicrobial and each microorganism evaluated, as shown in Table 2. Because MIC values may vary depending on the bacterial strain, experimental conditions, and assay methodology, these baseline values were experimentally confirmed before evaluating the ternary combinations. These experimentally confirmed MIC values were subsequently used to define the concentration ranges evaluated in the ternary mixtures.

Table 3 presents the combinations used to generate antimicrobial mixtures according to the chessboard design. When the concentration of either natural antimicrobial (A or B) was zero, those formulations resulted in a binary mixture with potassium sorbate; these were not evaluated in this study. Since one of the main objectives of this work was to identify synergistic conditions, only true ternary mixtures containing potassium sorbate and both natural compounds were considered. Accordingly, combinations located above the line of additivity, which represent true ternary systems involving potassium sorbate and both natural compounds, were selected for evaluation. The tested ternary mixtures for both microorganisms at the evaluated pH values are presented in Tables S1 and S2 (Supplementary Material).

### 2.3. Determination of the Fractional Inhibitory Concentration (FIC)

Model solid systems were prepared using TSA as the base. The water activity in the medium was adjusted to 0.99 by adding sodium chloride. The medium was sterilized at 121 °C for 15 min and cooled. Then the pH was adjusted to 4.5 or 5.5 using a 1 N hydrochloric acid solution (Merck Co., Darmstadt, Germany), sterilized by filtration through a 0.45 µm cellulose membrane (Micro Filtration Systems, Dublin, CA, USA).

Alcoholic solutions (5%, *v*/*v*) of thymol, vanillin, eugenol, carvacrol, and citral, as well as aqueous potassium sorbate solutions (5%, *v*/*v*), were prepared. The solutions were sterilized by filtration through a 0.45-µm cellulose membrane (Micro Filtration Systems, Dublin, CA, USA) and stored at 4 °C in amber bottles. For each formulation, the two natural antimicrobial compounds were added to the model systems, which were then cooled and acidified. The volume of each solution added to the agar medium was adjusted to obtain the concentrations evaluated in the ternary mixtures. Control samples without antimicrobials were also prepared. To ensure uniform distribution of the lipophilic molecules throughout the aqueous phase, the mixtures were maintained under continuous magnetic stirring at a controlled temperature immediately prior to pouring. Finally, the agar medium was poured into 15-cm Petri dishes and allowed to solidify.

**Figure 1:**
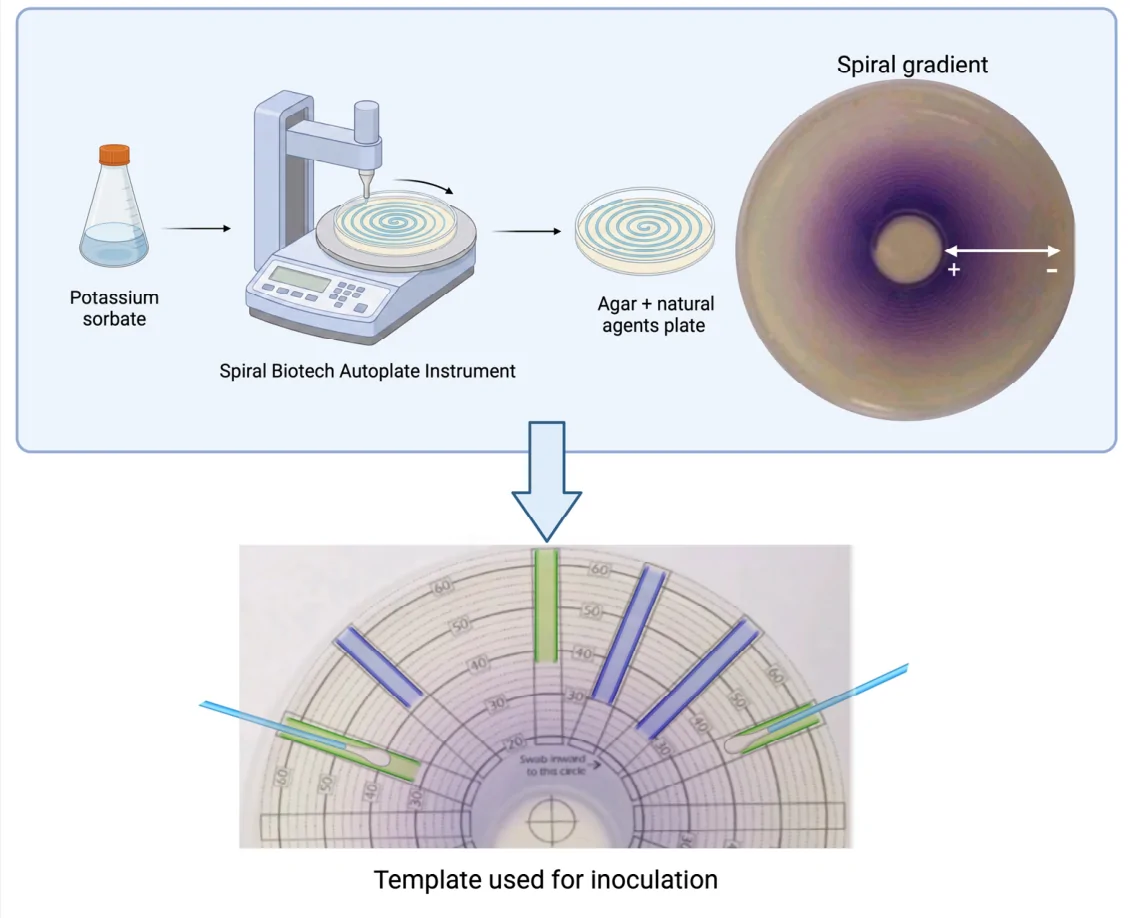
Procedure and template used for potassium sorbate deposition and bacterial inoculation: the concentric lines describe the path of the spirally deposited antimicrobial solution, and each radial line indicates where the inoculum is deposited.

The Spiral Gradient Method (Autoplate 4000, Spiral Biotech, Norwood, MA, USA) was used to deposit 50 µL of antimicrobials in an exponential spiral pattern, establishing a continuous concentration gradient on a single agar plate. Quantification of potassium sorbate MICs using the SGE v. 1.3 software (Spiral Biotech, Norwood, MA, USA) depends strictly on detecting an inhibition zone (gradient endpoint) along the radial inoculation line (Figure 1). The antimicrobial was then allowed to diffuse into the agar for two hours under aseptic conditions. Triplicate plates for each system were inoculated using a sterile swab that was moistened in TSB containing the bacterial culture. The inoculum was applied to the agar surface in a linear streaking pattern from the center to the edge of the plate with a standardized inoculum of *Listeria innocua* and *Salmonella* Typhimurium (≈10^8^ CFU/mL), as illustrated in Figure 1. The inoculated plates containing *Listeria* and *Salmonella* were incubated at 35 °C for 48 h, and then their growth was measured using the template shown in Figure 1.

Based on the resulting measurements, mean values were calculated, and the MIC of potassium sorbate was determined for each microorganism using the Spiral Gradient Endpoint v. 1.3 software (Spiral Biotech, Inc., Norwood, MA, USA). Although using a 5% (*v*/*v*) ethanol vehicle and continuous magnetic stirring likely improved the initial dispersion of hydrophobic compounds, water solubility still presents a challenge for this setup. Localized partitioning of lipophilic monoterpenes in the agar may influence the accuracy of the visually registered growth endpoints. As a result, the calculated values should be regarded as approximate indicators of interaction within this solid-matrix model. In those inoculated plates where the combination of potassium sorbate and the subinhibitory natural antimicrobials resulted in bacterial growth extending completely to the center of the Petri dish, no endpoint could be recorded within the maximum concentration range deposited by the spiral plater; the software was unable to calculate numerical values for these formulations. Therefore, these combinations were classified as ‘no inhibition detected under tested conditions’ and were excluded from the analysis required for numerical data.

To determine whether the mixtures exhibit synergism, additivity, or antagonism, the inhibitory concentration data for each antimicrobial mixture were converted to fractional inhibitory concentrations (FICs) using the methodology reported by López-Malo et al. [36]:

$$FIC_{i}=\frac{MIC_{i}^{\left(combo\right)}}{MIC_{i}^{\left(single\right)}},\;\;\;i\in \{A,B,C\}$$

where $MIC_{i}^{\left(combo\right)}$ denotes the MIC of the component i in the presence of the other two agents in the mixture, and $MIC_{i}^{\left(single\right)}$ represents the MIC for agent *i* when tested individually.

A fractional inhibitory concentration index (FICI) of 1 indicates an additive effect, meaning that the concentrations of antimicrobial agents required to inhibit microbial growth in combination are equivalent to the sum of their expected effects based on their individual MIC values. Antimicrobial synergism is typically defined by FIC index values ≤ 0.5, whereas values between 0.5 and 1 indicate additivity or indifference. The antimicrobial mixture FIC index is computed as the sum of the individual FICs:

$$FICI=\;FIC_{A}+\;FIC_{B}+\;FIC_{C}$$

The FICI is widely used to evaluate antimicrobial interactions in multi-agent systems, allowing the assessment of whether combinations enhance, preserve, or reduce the expected activity of individual components. It is derived from Loewe’s additivity principle, which describes non-interactive behavior as the sum of fractional inhibitory contributions of each agent relative to its individual MIC [31]. In this framework, the FICI provides a scalar approximation of deviations from additivity in multidimensional interaction spaces.

Calculation of the FICI yields a single quantitative value used to classify antimicrobial interactions as additive, synergistic, or antagonistic. According to the classical Loewe additivity framework, combinations are considered additive when FICI ≈ 1, synergistic when FICI < 1, and antagonistic when FICI > 1. This approach can be extended from pairwise interactions to higher-order systems, including ternary mixtures and multidimensional experimental designs [38]. Its relevance lies in its ability to identify effective combinations that may reduce required dosages and improve antimicrobial performance.

Interpretation thresholds may vary across studies; however, a commonly used classification defines FICI ≤ 0.5 as synergism, 0.5–1.0 as additivity or indifference, and >1 as antagonism [39,40,41]. The statistical analysis focused on ternary formulations with FICI values below 1.0 under the evaluated conditions. Given the methodology used to determine the concentrations of the agents in the mixture that inhibited bacterial growth, the results presented in this study correspond exclusively to combinations exhibiting additive and synergistic interactions, according to the adopted FICI classification scheme. Formulations meeting this criterion were retained for subsequent interpretation and included in all tables, figures, and multivariate analyses.

### 2.4. Statistical Analysis

Statistical analyses were performed using Minitab v. 22.5.1 software (Minitab LLC, State College, PA, USA), and statistical significance was assessed at the 95% confidence level. Principal component analysis (PCA) was applied to explore similarities among antimicrobial formulations and experimental conditions. The analysis included the MIC and FICI values obtained against *Listeria innocua* and *Salmonella* at pH 4.5 and 5.5. Prior to PCA, all variables were mean-centered and autoscaled (unit variance) to eliminate differences in measurement scale. The number of principal components retained was selected based on the cumulative explained variance and the scree plot. Score plots were used to identify clustering patterns among formulations, whereas loading plots were examined to determine the variables contributing most strongly to the observed variability.

Partial least squares (PLS) regression was performed to investigate the relationship between antimicrobial formulations and inhibitory performance. The predictor matrix (X) consisted of the concentrations of potassium sorbate and the natural antimicrobial compounds, together with the experimental conditions (pH and target microorganism). The response matrix (Y) included either the FICI or the total MIC for each formulation. Prior to PLS regression, pH was converted into a coded predictor variable (4.5 = 1 and 5.5 = 0). This coding strategy was implemented to balance the operational matrices and minimize scale-dependent biases between environmental and concentration metrics.

The optimal number of latent variables was determined through cross-validation procedures. Model performance was evaluated using the coefficient of determination (R^2^) and the root mean square error (RMSE). Variable importance (standardized coefficients) was utilized to identify the antimicrobial compounds and experimental factors that exert the strongest influence on antimicrobial synergism and inhibition efficacy.

## 3. Results and Discussion

The tables, figures, and multivariate analyses presented in this study show the formulations that exhibited synergistic or additive interactions (FICI ≤ 1.0). Given the methodology used in the study, no combinations with FICI values greater than 1.0 (indicative of non-synergistic interactions) were detected. This approach allowed the discussion to focus on preservative systems with the greatest potential to reduce antimicrobial concentrations without compromising inhibitory efficacy.

**Figure 2:**
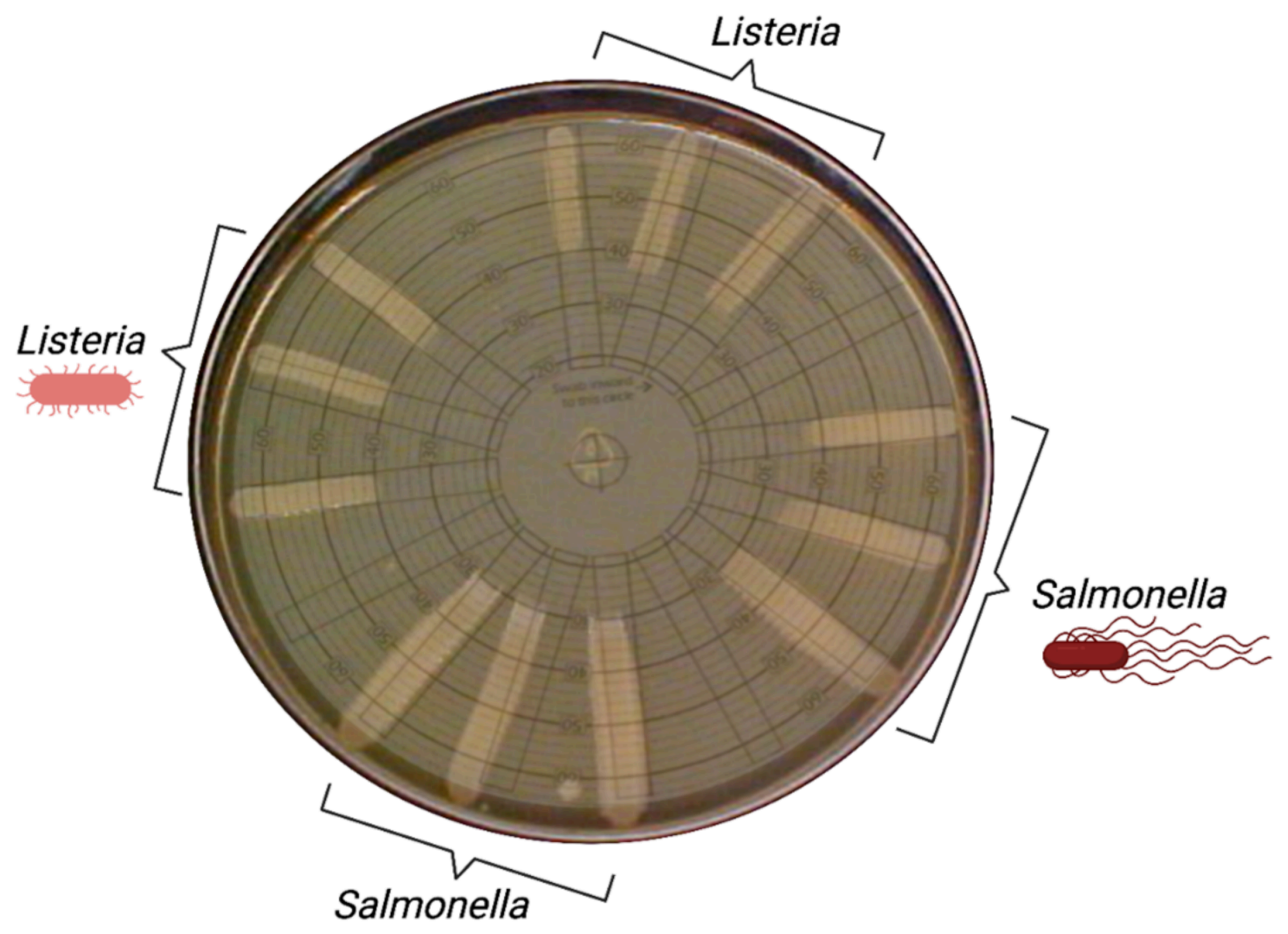
Inhibition profile of *Listeria innocua* ATCC 51742 and *Salmonella* Typhimurium ATCC 14028 on tryptic soy agar (pH 4.5) supplemented with a mixture of carvacrol and thymol. The bacterial growth limit visually defines the minimum inhibitory concentration of potassium sorbate (deposited in a spiral gradient) under these conditions.

**Figure 3:**
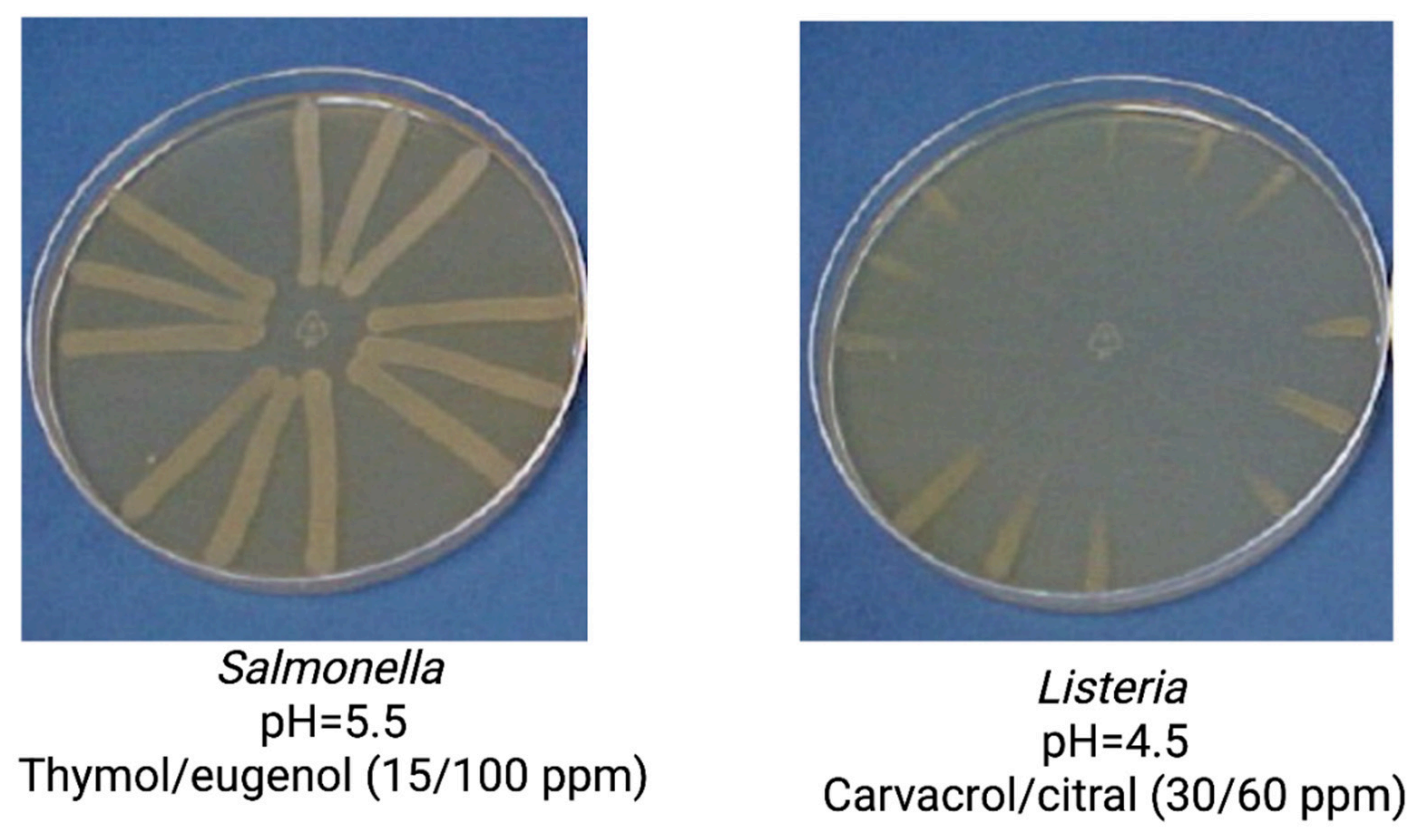
Inhibition profile of *Listeria innocua* ATCC 51742 and *Salmonella* Typhimurium ATCC 14028 on tryptic soy agar (pH 4.5 or 5.5) supplemented with a mixture of thymol-eugenol or carvacrol-citral. The bacterial growth limit visually defines the minimum inhibitory concentration of potassium sorbate (deposited in a spiral gradient) under these conditions.

Figure 2 and Figure 3 illustrate the application of the spiral gradient plating method to evaluate the combined effects of potassium sorbate, pH, and two natural antimicrobial compounds on the growth of *Listeria innocua* and *Salmonella*.

The spiral plating system generated a continuous concentration gradient of potassium sorbate across the agar surface while maintaining constant concentrations of the natural antimicrobials, allowing the inhibitory concentration of potassium sorbate to be determined directly from the point at which bacterial growth ceased. The sharp transition between confluent growth and complete inhibition enabled accurate determination of the MIC under each experimental condition while substantially reducing the number of assays required compared with conventional dilution methods.

Among the ternary formulations evaluated (Table 4), which focus exclusively on synergistic interactions (FICI < 0.50), FICI values ranged from 0.252 to 0.496, while the remaining additive-tested ternary mixtures were transferred to the Supplementary Material (Tables S1 and S2). Synergistic mixtures were achieved across both pH and bacterial targets, particularly when including isomeric thymol and carvacrol or vanillin with potassium sorbate. The addition of potassium sorbate reduced the concentrations of the natural compounds required to inhibit bacterial growth. For *Listeria innocua* at pH 4.5, the presence of potassium sorbate resulted in natural compound reductions ranging from 62.9% to 97.4%, reducing antimicrobial agents like thymol and carvacrol at concentrations as low as 15 ppm to 30 ppm to inhibit bacterial growth. A similar pattern was observed for *Salmonella* Typhimurium at pH 4.5, where natural compound concentrations were reduced by 76.3% to 90.0% in the presence of sorbate. This dose-reduction behavior demonstrates a hurdle effect in which natural agents and conventional preservatives act through multi-target mechanisms [42]. Many of the tested formulations (Table 4) substantially reduce the doses of both the conventional preservative and the natural compounds required to maintain antimicrobial efficacy.

Similar interaction patterns were observed across all conditions, although the magnitude of the synergistic response varied with both the target microorganism and the medium pH. Overall, lower pH enhanced antimicrobial activity, resulting in lower MIC and FICI values for several formulations. Evaluating each formulation under multiple environmental conditions enabled the identification of preservative systems that remained effective across diverse microbial and pH conditions.

The enhanced antimicrobial activity observed in thymol-carvacrol mixtures is consistent with proposed mechanisms involving cytoplasmic membrane disruption, increased membrane permeability [34], and the facilitation of intracellular accumulation of weak-acid preservatives such as potassium sorbate [23]. Vanillin has also been reported to impair membrane integrity and cellular metabolism [35], potentially further potentiating sorbate’s inhibitory effect. These complementary mechanisms likely explain the synergistic interactions observed in several ternary formulations, supporting the use of combined preservative systems to enhance antimicrobial efficacy while reducing the concentration of individual additives. Moon & Rhee [43] evaluated a carvacrol and thymol mixture (1 mM, 0.0157%) and reported that it induced the greatest antibacterial activity (*E. coli* O157:H7, *S.* Typhimurium, *L. monocytogenes*) in soy sauce; in semi-skim milk, cinnamon EO and vanillin exert bactericidal activity against *L. monocytogenes* and *E. coli* [44]; ternary mixtures (vanillin, citral, and potassium sorbate) showed antibacterial activity in fruit purées (mango and melon) [30]; carvacrol-eugenol and thymol-eugenol mixtures in water rinses decontaminated (*E. coli* O157:H7) fresh-cut Iceberg lettuce when used as sanitized solutions [45]. Ribes et al. [46] evaluated the antimicrobial activity (against *E. coli*) of eugenol and vanillin immobilized in MCM-41 microparticles; bacteria were inhibited in apple and grape juices. In selected studies, sensory scores were maintained after mixtures were incorporated into food, for example, in soy sauce [43]. The key point in using EO components in food applications is their flavor/odor compatibility with food. For instance, in the present work, mixtures of citral-vanillin-PS can be suggested in dessert recipes, and thymol-carvacrol-potassium sorbate for poultry/meat or poultry/meat preparations.

Table 4 presents the concentrations of ternary mixtures comprising two natural antimicrobials and potassium sorbate that effectively inhibited *Listeria* and *Salmonella* (FICI < 0.5) at pH 4.5 and 5.5. The pH influenced the antimicrobial activity of potassium sorbate, a weak-acid preservative, which increases as more sorbic acid remains undissociated. At pH 4.5, a greater proportion of sorbate is present in its undissociated form, facilitating its diffusion across the bacterial membrane and leading to intracellular acidification. Several formulations had lower MIC and FICI at pH 4.5 than at pH 5.5, indicating that acidic conditions enhance sorbate’s cooperative effects and efficacy. This aligns with previous studies showing better sorbate performance at lower pH and highlights the effect of environmental conditions on preservatives [47]. Furthermore, these formulations reduce the use of synthetic preservatives, showing significant reductions in the percentage of potassium sorbate (>70%) required in the mixture compared with its use as a single agent. In most of the selected mixtures, potassium sorbate was reduced by more than 90%. When observing the total reduction in antimicrobial agents or natural compounds (Table 4), it is also observed that reductions greater than 70% can be achieved, depending on the mixture chosen, the pH, and the type of microorganism.

Differences were also observed between microorganisms. In general, *Listeria innocua* exhibited lower MIC and FICI values than *Salmonella* Typhimurium, indicating greater susceptibility to the antimicrobial mixtures. This behavior is consistent with the structural differences between Gram-positive and Gram-negative bacteria. It is reported that the outer membrane of Gram-negative bacteria may act as an additional permeability barrier that restricts the diffusion of hydrophobic compounds such as thymol, carvacrol, citral, and eugenol, thereby reducing antimicrobial efficacy [48,49,50,51]. Nevertheless, several ternary systems maintained synergistic interactions against both microorganisms, suggesting that combining sorbate with membrane-active natural compounds may partially overcome this barrier effect.

Among the evaluated systems, mixtures containing carvacrol and thymol consistently generated low FICI values across conditions, highlighting the major contribution of phenolic monoterpenes to the observed antimicrobial synergism. According to previous studies, both isomeric monoterpenes can exert potent antibacterial activity by incorporating into the lipophilic phase of the cytoplasmic membrane, which can alter structural integrity and proton gradients, as well as increase membrane permeability [52,53,54,55,56,57]. This membrane-permeabilizing effect is hypothesized to lower the intracellular kinetic barriers, thereby facilitating the influx and subsequent cytoplasmic action of potassium sorbate and other co-administered antimicrobial agents [56,58]. Vanillin-containing mixtures also yielded low FICI values, despite vanillin exhibiting significantly lower potency when evaluated individually. This behavioral discrepancy underscores that antimicrobial performance in ternary systems is not merely a reflection of the intrinsic activity of individual constituents, but rather depends on complementary, multi-target mechanisms of action [44,57]. Specifically, previous studies indicated that while monoterpenes may destabilize the cell envelope, milder structures such as phenolic aldehydes like vanillin can easily penetrate and disrupt internal cellular targets, such as enzymatic pathways or ATP metabolism, resulting in a pronounced, cooperative interaction [59].

Collectively, the results demonstrate that a complex interplay between antimicrobial composition, environmental pH, and microbial physiology governs the efficacy of ternary preservative systems. The observation of similar interaction patterns across experimental conditions, together with condition-dependent differences in the magnitude of synergism or additivity, supports the use of multivariate approaches. Consequently, the PCA and PLS models were constructed exclusively from formulations exhibiting favorable antimicrobial interactions (FICI < 1.0), ensuring that the multivariate analyses reflected patterns associated with effective preservative combinations rather than non-interactive systems.

**Figure 4:**
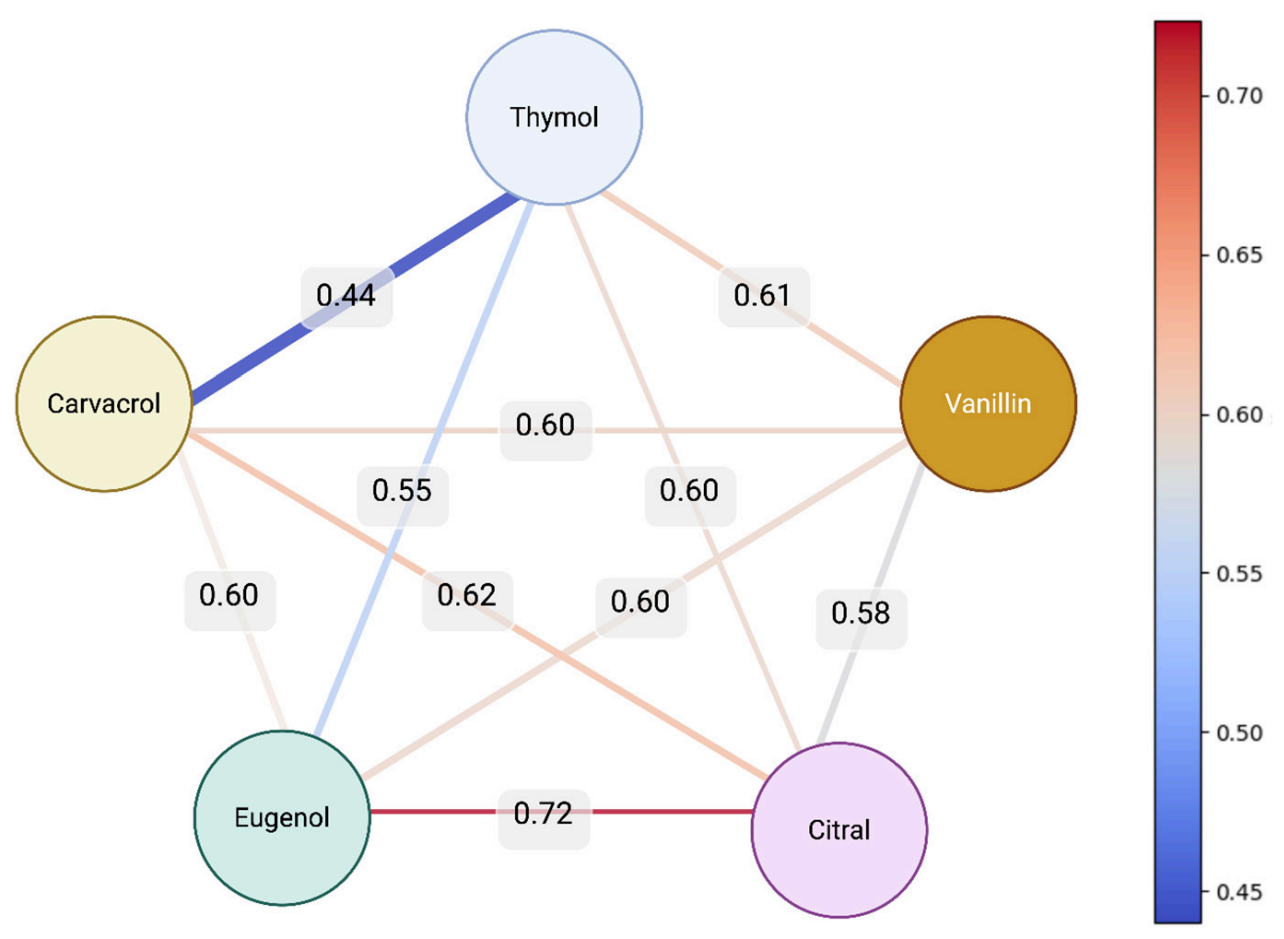
Heat map of the average fractional inhibitory concentration index (FICI) values of the ternary mixtures evaluated in the 4 cases (pH 4.5 and 5.5, and against *Listeria innocua* ATCC 51742 and *Salmonella* Typhimurium ATCC 14028); all mixtures include potassium sorbate as the third compound.

Figure 4 summarizes the overall behavior of the ternary mixtures, with each edge representing the average FICI for formulations containing potassium sorbate and two natural antimicrobials across all four evaluated conditions (*Listeria* and *Salmonella* at pH 4.5 and 5.5). Lower FICI values indicate a more favorable interaction (synergism), while higher values reflect less cooperation between the compounds (additivity). The average FICI values ranged from 0.44 to 0.72; the combination with the lowest average FICI was thymol–carvacrol (FICI = 0.44), while the combination with the highest average FICI was eugenol–citral (FICI = 0.72). The color gradient and the thickness of the connections allow for quick visualization of these differences, highlighting the more and less favorable associations. In the thymol-carvacrol combination, both compounds are structurally related phenolic monoterpenes; thus, their reported mechanisms of action are similar and primarily involve alterations to the cytoplasmic membrane, loss of membrane potential, leakage of intracellular components, and disruption of cellular homeostasis [52,54]. The observed synergism suggests that the simultaneous presence of both compounds enhances these effects and promotes the complementary action of the sorbate. Conversely, the eugenol-citral combination showed the highest average FICI value (0.72), indicating a less efficient interaction. Although both compounds possess recognized antimicrobial activity, the results suggest their mechanisms of action may not complement each other as effectively as those of phenolic compounds, or they may compete for similar interaction sites on the bacterial cell, thus reducing the benefit of the combination. The other pairs showed relatively close intermediate values (0.55–0.62), suggesting that most mixtures generate comparable levels of antimicrobial cooperation. An interesting aspect illustrated in Figure 4 is that phenolic compounds (thymol, carvacrol, and eugenol) appear in several combinations with the lowest average FICI values. This aligns with numerous studies indicating that phenols derived from EOs are particularly effective at increasing cell permeability and facilitating the entry of other antimicrobials, thus promoting synergistic interactions [48,60]. Furthermore, although vanillin typically exhibits lower individual or baseline antimicrobial activity than potent monoterpenes such as thymol or carvacrol, its multi-component combinations yielded average FICI values comparable to those obtained with more active compounds. This phenomenon highlights that a phytocompound’s net contribution to a preservation matrix is not strictly a function of its autonomous potency, but rather depends on its ability to complement non-competitive, distinct mechanisms of action within a multi-hurdle system [42]. While carvacrol and thymol act as strong bactericidal agents that may interact with the lipid bilayer, disrupting membrane fluidity and causing rapid intracellular leakage, vanillin may act primarily as a bacteriostatic phenolic aldehyde [51]. It has been reported that vanillin acts predominantly on internal cellular systems, ionic gradients, cellular respiration, and cell division pathways [59,61]. When combined, carvacrol and thymol may alter membrane permeability, which facilitates the passive influx of vanillin into the cytoplasm, where it can reach metabolic targets [44]. Citral may interact with cell membranes and intracellular enzymes, disrupting cellular integrity [62]. Eugenol’s amphiphilic nature may allow it to insert into lipid bilayers and inhibit cytoplasmic enzymes like ATPase [63]. Khwaza et al. [15] combined diverse compounds—phenolic aldehydes, monoterpene aldehydes, allylbenzene phenolics, and isomeric monoterpene phenols—that target multiple bacterial cell structures and pathways simultaneously, enhancing their antimicrobial activity. This prevents saturation or displacement at lipophilic receptors, avoiding antagonism seen in pure isomeric mixtures. Our findings show that less potent compounds like vanillin, combined with multi-target agents like citral, eugenol, thymol, and carvacrol, serve as effective adjuvants. They achieve synergism and reduce the total agent amount in the ternary mixture.

### 3.1. Principal Component Analysis

**Figure 5:**
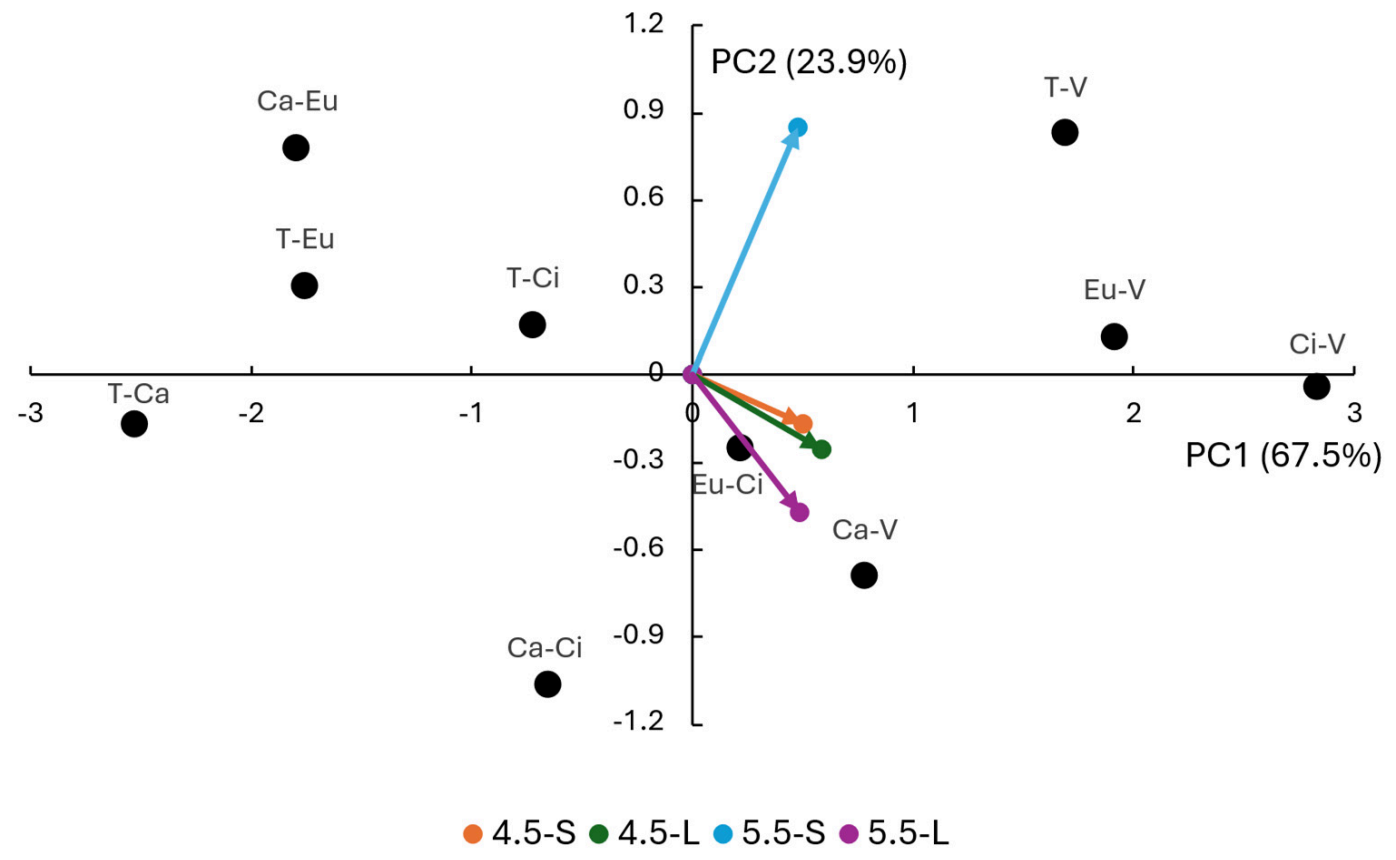
Biplot of the principal component analysis (PCA) for the total minimum inhibitory concentration (MIC) values of the ternary antimicrobial mixtures. The black dots represent the scores of the combinations of two natural antimicrobials and potassium sorbate [Ca: carvacrol, T: thymol, Eu: eugenol, Ci: citral, V: vanillin]. The colored vectors represent the loadings for the four experimental conditions evaluated by pH and microorganism (S: *Salmonella* Typhimurium ATCC 14028; L: *Listeria innocua* ATCC 51742). Orange and green lines represent *Salmonella* and *Listeria* at pH 4.5, respectively. Blue and purple lines represent *Salmonella* and *Listeria* at pH 5.5, respectively.

The principal component analysis biplot (Figure 5) explains 91.4% of data variability. PC1 (67.5%) mainly separates mixtures by antimicrobial efficacy, while PC2 (23.9%) shows differences in microorganism responses based on medium pH. The spatial separation along the PC1 efficiency axis aligns directly with individual MIC values of the single compounds. The isomeric monoterpene phenols, thymol and carvacrol, which have the lowest individual MIC, strongly load onto the negative quadrant of PC1. Consequently, multi-component formulations containing these high-potency fractions are pulled toward the negative region of PC1. In contrast, compounds with higher baseline individual MIC values, such as vanillin, load onto the positive axis of PC1. This division explains why mixtures containing elevated fractions of vanillin migrate toward positive PC1 scores, indicating a quantitative increase in the required MIC.

The vectors corresponding to the pH 4.5 conditions (4.5-S and 4.5-L) show very similar projections and magnitudes, with positive charges in PC1 and slightly negative charges in PC2. This displays that at an acidic pH, the inhibitory behavior of the ternary mixtures is homogeneous and independent of the microorganism being evaluated. In contrast, at pH 5.5, the charges for *Salmonella* (5.5-S, positive PC2) and *Listeria* (5.5-L, negative PC2) are markedly separated along the vertical axis (PC2), evidencing a microorganism-dependent effect as acidity decreases. The T-Ca (Thymol-Carvacrol), T-Eu (Thymol-Eugenol), and Ca-Eu (Carvacrol-Eugenol) combinations were located at the negative end of PC1, in the opposite direction to all the variable vectors. Because the vectors point toward higher MIC values, the positions of these three mixtures on the negative PC1 indicate that they have the lowest total MIC values, establishing them as the combinations with the greatest overall inhibitory potency. Conversely, the mixtures formulated with vanillin (Ci-V, Eu-V, T-V, and Ca-V) showed positive scores in PC1, aligning with the vector direction. This confirms that including vanillin increases the total required MIC.

**Figure 6:**
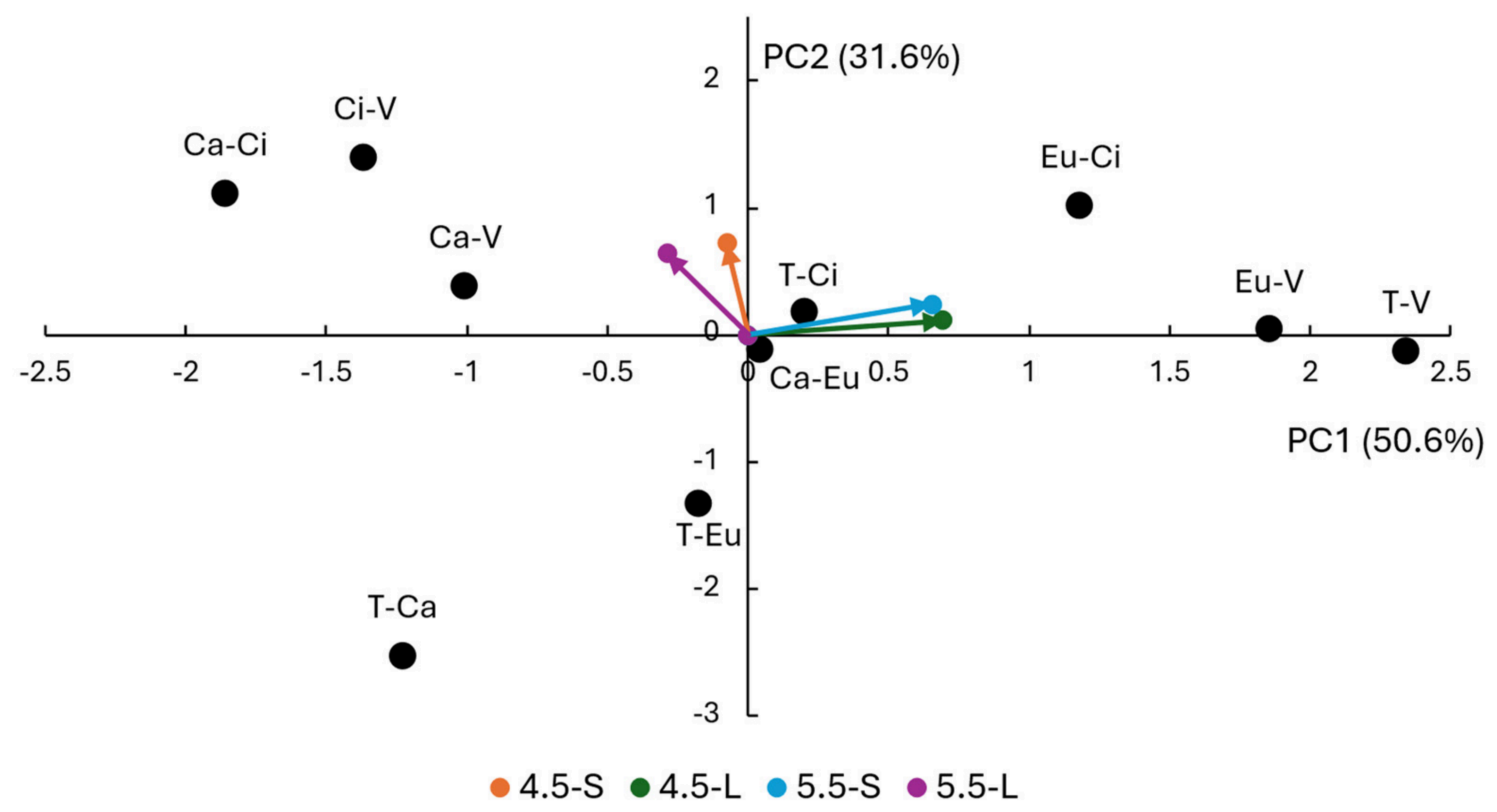
Biplot of the principal component analysis (PCA) for the fractional inhibitory concentration indices (FICI) of the ternary antimicrobial mixtures. The black dots represent the scores of the combinations of two natural antimicrobials and potassium sorbate [Ca: carvacrol, T: thymol, Eu: eugenol, Ci: citral, V: vanillin]. The colored vectors represent the loadings for the four experimental conditions evaluated by pH and microorganism (S: *Salmonella* Typhimurium ATCC 14028; L: *Listeria innocua* ATCC 51742). Orange and green lines represent *Salmonella* and *Listeria* at pH 4.5, respectively. Blue and purple lines represent *Salmonella* and *Listeria* at pH 5.5, respectively.

In the FICI biplot (Figure 6), the first two principal components explain 82.2% of the cumulative variance. PC1 (50.6%) describes the transition from cooperative or synergistic interactions (negative values) to indifference or loss of synergism (positive values). PC2 (31.6%) captures interaction specificity as a function of the combination of pH and microorganism. A strong positive correlation is observed between the vectors of conditions 4.5-L and 5.5-S, which share positive charges in both PC1 and PC2 (oriented towards the upper-right quadrant). This indicates that the way ternary mixtures interact with *Listeria* at pH 4.5 is similar to their response against *Salmonella* at pH 5.5. Additionally, the 4.5-S condition mainly aligns with the positive axis of PC2, with PC1 near zero, indicating that it performs independently of the other conditions.

The T-Ca (Thymol-Carvacrol) and T-Eu (Thymol-Eugenol) combinations were located with negative scores on PC2 and negative or near-zero values on PC1, placing them in the quadrant opposite to the influence of the response vectors. Their distance from areas with high FICI values is statistically confirmed, showing that these two combinations exhibit the lowest FICIs in the study and indicate synergistic or additive effects under the evaluated scenarios. The Ca-Ci, Ci-V, and Ca-V combinations showed negative scores on PC1, directly opposing the 4.5-L and 5.5-S vectors, indicating good interactive performance specific to these two situations. Finally, mixtures such as Eu-V and T-V shifted toward the highest and most positive PC1 values, consistent with the variable loadings, statistically indicating an increase in the FICI promoted by the addition of vanillin.

The integration of both principal component analyses reveals that the ternary mixtures formulated with thymol-carvacrol (T-Ca) and thymol-eugenol (T-Eu) are the most promising options for controlling both microorganisms. The MIC PCA demonstrates that these combinations possess the highest absolute inhibitory potency (lowest total MIC values), which consistently coincides with the FICI PCA, where they are positioned as the mixtures with the lowest indices, confirming a strongly cooperative or synergistic interaction. From a mechanistic perspective, the synergism between these compounds has been attributed to high hydrophobicity; thymol and carvacrol can act directly on the bacterial cytoplasmic membrane, altering its permeability and causing the loss of essential ions such as potassium [58]. This structural damage can dissipate the pH gradient essential for cellular homeostasis [64]. Additionally, in previous reports, co-administration of these molecules has been shown to inhibit efflux pumps and alter bacterial ATP metabolism [65], thereby facilitating entry of potassium sorbate into the cell [13]. Conversely, the addition of vanillin is counterproductive in both cases, as it shifts their respective mixtures toward positive scores on PC1 in both graphs, which simultaneously results in an increase in the required MIC and a loss of synergism, suggesting possible structural interference or competition for membrane binding sites. Vanillin is effective in low doses in multi-hurdle systems, but excess can cause antagonism. Lipophilic monoterpenes like thymol and carvacrol may disrupt membranes by accumulating in the lipid bilayer [51]. However, it is hypothesized that vanillin’s phenolic aldehyde structure can, in high concentrations, hinder or compete with these monoterpenes for hydrophobic membrane access [66], potentially blocking membrane permeabilization and reducing their bactericidal effects [61]. The acidic pH (4.5) causes a uniform biological response in both *Listeria* and *Salmonella*, probably due to proton-related cell stress. Raising the pH to 5.5 triggers specific responses that depend on the microorganism, which influence effective concentration in the mixture.

### 3.2. Partial Least Squares (PLS) Analysis

Partial least squares (PLS) regression was performed using Minitab software (v. 22.5.1) to investigate the relationship between the antimicrobial formulation components and their inhibitory performance. The predictor matrix (X) consisted of the concentrations of potassium sorbate and the natural antimicrobial compounds (Vanillin, Citral, Eugenol, Thymol, and Carvacrol), together with the pH and target microorganism. The response matrix (Y) evaluated two independent biological endpoints: the FICI and the total MIC of each formulation.

The analysis was executed on a robust dataset comprising N = 125 independent observations. To prevent overfitting and ensure model generalizability, cross-validation was conducted via the Leave-One-Out procedure. Under this iterative mechanism, the optimal number of latent variables (components) was determined by maximizing the Predicted R^2^ and minimizing the Predicted Residual Error Sum of Squares (PRESS) and the Root Mean Square Error of Cross-Validation, which assess model performance solely on out-of-sample data points withheld during calibration. Model optimization was guided by a parsimonious selection approach, considering both the contribution of individual predictors to statistical noise reduction and the incremental behavior of the model’s predictive power across latent dimensions. Model fit and stability were evaluated using the standard coefficient of determination (R^2^). Finally, variable importance was assessed through the analysis of standardized regression coefficients to isolate and identify the specific antimicrobial compounds and factors exerting the strongest direct or synergistic influence on both antimicrobial efficacy responses.

Analysis of the standardized PLS regression coefficients enabled the ranking and prioritization of the relative impact of each predictor variable on the system’s overall inhibitory capacity (Table 5), an approach widely validated for interpreting the relative weight of predictors regardless of their original metrics [67,68]. Dai et al. [69] investigated yeast inhibition with three-agent combinations of lauric arginate, cinnamic acid, and either sodium benzoate or potassium sorbate. They developed a model predicting critical concentrations of sodium benzoate or potassium sorbate based on levels of lauric arginate and cinnamic acid, demonstrating that these combinations were highly effective at pH 3.0. The model also facilitated cost comparison to identify the most economical options.

The analysis used out-of-sample Leave-One-Out cross-validation to ensure genuine prediction, avoiding reliance on standard fitness parameters that can be overinterpreted due to the link between formulation concentrations and responses. The standardized coefficients for the FICI model demonstrated that the formulation components exert primary control over synergism. Potassium sorbate appeared as the dominant predictor with the highest positive weight (+0.8736), followed in descending order by eugenol (+0.7757), carvacrol (+0.6206), citral (+0.5119), thymol (+0.4896), and vanillin (+0.3950). These positive standardized coefficients indicate that increasing these individual components by one standard deviation within the formulation space drives the response toward higher FICI thresholds, systematically shifting the system away from the optimal synergistic zone (FICI < 0.5) [48,49]. Consequently, maximum synergistic efficiency is mathematically and biologically restricted to specific experimental regions characterized by lower fractional concentrations of the antimicrobials tested. This behavior suggests that minimizing individual agent fractions may prevent cellular saturation kinetics, or competitive displacement at outer-membrane or cytoplasmic targets, a phenomenon previously documented when hydrophobic plant volatiles partition into structural membrane lipids [36,50,51].

The bacterial species variable (*Listeria* and *Salmonella*) yielded the lowest standardized coefficient magnitude. This minor impact suggests that the efficacy of the formulated ternary mixtures is robust across the tested targets. However, it is important to acknowledge that only one strain per species was evaluated. Given the well-documented intraspecific variability in stress tolerance and inhibition among foodborne pathogens [70], further studies incorporating multiple strains are required to confirm this uniform effectiveness at a broader species level.

The standardized coefficients (Table 5) for the total MIC model revealed a shift in the hierarchy of predictor importance compared to the FICI analysis. Potassium sorbate remained the most critical predictor, with a robust coefficient of +0.4701, followed by vanillin, which gained prominence as a secondary predictor with a standardized coefficient of +0.4492. The positive values of both coefficients indicate that their inclusion at high proportions directly increases the total absolute concentration required to inhibit microbial growth. For vanillin, this behavior suggests that, although its synergistic interaction is discrete and has a minor impact on the FICI metric, its molecular weight has a strong relative influence on the total quantity of the ternary formulation.

The pH parameter showed a positive standardized coefficient, indicating that increasing pH from 4.5 to 5.5 slightly increases the absolute total dose of the mixture required for inhibition. Thymol and carvacrol exhibited standardized coefficients of practically negligible magnitude. The type of bacteria (non-significant coefficient) confirms that the absolute amount of the ternary mixture required to inhibit bacterial growth operates with uniform technical effectiveness for both Gram-positive *Listeria* and Gram-negative *Salmonella*.

The apparent multi-species effectiveness and cooperative performance, mathematically validated by PLS models, are consistent with recent literature exploring volatile monoterpenes and phenolic compounds as potent antimicrobial agents [53]. Specifically, carvacrol and thymol—despite being structural isomers—exhibit distinct interaction behaviors and distinct FICI trajectories depending on the design of the delivery system [53]. These natural agents may compromise bacterial viability through simultaneous cellular interferences [53,54]. This multi-targeted disruption structurally prevents cell recovery, successfully minimizing the MIC required for total inhibition [55]. Furthermore, the absence of mutual antagonism among eugenol, thymol, and carvacrol highlights their compatibility in multi-component mixtures, where determining the minimum effective concentrations is essential for optimizing industrial applications [56]. Individual natural compounds often fail to exert strong bactericidal effects in isolation; incorporating them into multi-ingredient or complex formulations unlocks powerful, synergistic interactions that can eliminate pathogens across diverse environmental conditions [57]. Therefore, the low FICI and MIC values, statistically verified by our PLS analysis, are supported by reported work showing that these natural and synthetic mixtures can simultaneously destabilize key cellular structures without triggering competitive interference at potential shared sites of action.

## 4. Conclusions

This study demonstrated that ternary preservative systems combining potassium sorbate with natural antimicrobial compounds can effectively inhibit *Listeria innocua* and *Salmonella* Typhimurium while substantially reducing the concentration of individual antimicrobial agents required for growth inhibition. Several formulations exhibited synergistic interactions, highlighting the potential of combining conventional preservatives with plant-derived compounds to develop lower-dose preservation strategies. Multivariate analyses provided complementary insight into the experimental dataset. Principal Component Analysis identified the major sources of variability among formulations and experimental conditions, whereas Partial Least Squares regression revealed the formulation variables most strongly associated with antimicrobial performance. Together, these approaches offered a robust framework for interpreting complex interaction patterns in multicomponent preservative systems. The results also demonstrated that the magnitude of antimicrobial synergism depended on both pH and the target microorganism, emphasizing the importance of considering environmental conditions when formulating preservatives. These findings provide a rational basis for selecting optimized ternary antimicrobial systems that combine high efficacy with reduced concentrations of synthetic preservatives. However, it must be emphasized that these antimicrobial systems were systematically evaluated under strict in vitro laboratory conditions, with the limitation that only one strain per species was tested. Therefore, their practical implementation will require further, comprehensive confirmation within representative food systems. Future research must validate these ternary matrices in real-world food scenarios to evaluate potential interferences and assess antimicrobial efficacy, sensory impact, and consumer acceptance under practical conditions. Such cross-validation remains essential for successfully translating these optimized preservative systems to commercial food applications.

## Figures and Tables

**Table 1:** Combinations of antimicrobials evaluated in the ternary mixtures.

Potassium sorbate	Thymol-carvacrol			
	Thymol-eugenol	Carvacrol-eugenol		
	Thymol-citral	Carvacrol-citral	Eugenol-citral	
	Thymol-vanillin	Carvacrol-vanillin	Eugenol-vanillin	Citral-vanillin

**Table 2:** Minimum inhibitory concentrations (ppm) for *Listeria innocua* ATCC 51742 and *Salmonella* Typhimurium ATCC 14028 at a_w_ 0.99, adjusted with NaCl and pH 4.5 or 5.5.

Antimicrobial	*Listeria innocua*		*Salmonella* Typhimurium	
	pH 4.5	pH 5.5	pH 4.5	pH 5.5
Potassium sorbate	500	600	500	600
Carvacrol	150	150	150	200
Citral	400	600	300	350
Vanillin	2700	3000	300	600
Thymol	150	200	150	250
Eugenol	200	250	250	250

**Table 3:** Chessboard design for assessing ternary mixtures with potassium sorbate and the antimicrobials A and B.

		Antimicrobial A				
		C1A	C2A	C3A	…	CnA
**Antimicrobial B**	C1B	C1A/C1B	C2A/C1B	C3A/C1B	…	CnA/C1B
	C2B	C1A/C2B	C2A/C2B	C3A/C2B	…	
	C3B	C1A/C3B	C2A/C3B	C3A/C3B		
	…	…	…			
	CnB	C1A/CnB				
	Binary mixtures					

**Table 4:** Concentrations (ppm), fractional inhibitory concentration index (FICI), minimal inhibitory sum of concentrations (MIC) of synergistic ternary mixtures on the inhibition of *Listeria innocua* ATCC 51742 at an a_w_ of 0.99 adjusted with NaCl and pH 4.5 or 5.5 on tryptic soy agar, and reduction (%) of the compounds in the ternary mixture.

		Antimicrobial Concentration (ppm)								Reduction (%)		
Bacteria	pH	Potassium Sorbate	Thymol	Carvacrol	Eugenol	Citral	Vanillin	FICI	MIC	Potassium Sorbate	Total	Natural Compounds
*Listeria innocua*	4.5	32	30				135	0.314	406	93.6	87.9	94.2
		32		30			135	0.314	278	93.6	91.7	94.2
		64				60	135	0.328	436	87.2	87.9	93.7
		64	15		30			0.378	158	87.2	81.4	87.1
		128		15			135	0.406	263	74.4	92.1	94.7
		128			20		135	0.406	278	74.4	91.8	94.7
		32		30		60		0.414	203	93.6	80.7	83.6
		128			20		180	0.423	303	74.4	91.1	93.1
		32	45			30		0.439	316	93.6	69.9	86.4
		64	45				45	0.445	203	87.2	93.9	96.8
		64		15		90		0.453	122	87.2	88.4	80.9
		128	15	15				0.456	158	74.4	80.3	90.0
		8	45			60		0.466	107	98.4	89.8	80.9
		128	30				45	0.473	263	74.4	92.1	97.4
		32	30			90		0.489	361	93.6	65.6	78.2
		128	30				90	0.489	154	74.4	95.4	95.8
		128			40		90	0.489	204	74.4	94.0	62.9
*Salmonella*	4.5	16	15	30				0.252	61	96.8	92.4	85.0
		32	15		40			0.324	87	93.6	91.7	86.3
		32	30		20			0.344	82	93.6	90.9	87.5
		64		30	20			0.408	114	87.2	87.3	87.5
		32		30	40			0.424	102	93.6	88.7	82.5
		64		30		30		0.428	124	87.2	86.9	86.7
		32		45	20			0.444	97	93.6	89.2	83.8
		128	15	15				0.456	158	74.4	80.3	90.0
		32	45			30		0.464	107	93.6	88.7	83.3
		32		45		30		0.464	107	93.6	88.7	83.3
		32		30		60		0.464	122	93.6	87.2	80.0
		32		15	80			0.484	127	93.6	85.9	76.3
		8		60	20			0.496	88	98.4	90.2	80.0
*Listeria innocua*	5.5	8			100		240	0.493	348	98.7	91.0	89.5
*Salmonella*	5.5	128	90				80	0.363	298	78.7	79.4	80.0
		8	90	20				0.473	118	98.7	88.8	75.6

**Table 5:** Unstandardized and standardized partial least squares (PLS) regression coefficients for the FICI and MIC predictive models.

Predictor Variable	FICI Model (5 Components)		MIC Model (3 Components)	
	Unstandardized	Standardized	Unstandardized	Standardized
Constant	−0.12874	0	−81.28	0
pH	0.1301	0.3146	46.6337	0.1997
Potassium Sorbate (ppm)	0.0865	0.8736	0.5238	0.4701
Carvacrol (ppm)	0.0052	0.6206	−0.3265	−0.0687
Citral (ppm)	0.0022	0.5119	0.5384	0.2192
Eugenol (ppm)	0.0039	0.7757	0.0454	0.0156
Vanillin (ppm)	0.0011	0.3950	0.7134	0.4492
Thymol (ppm)	0.0037	0.4896	−0.4656	−0.1074
Bacteria	0.0864	0.2418	Excluded	Excluded
	Model Fit Metrics			
	FICI Model (5 Components)		MIC Model (3 Components)	
LOO Cross-Validated R^2^ pred	60.75%		55.16%	
Standard Fit (R^2^)	68.16%		50.83%	
PRESS (Predicted Residual Error)	1.511		692,331	
Cumulative X Variance Explained	62.06%		45.55%	
Root Mean Square Error of Cross-Validation	0.11		74.42	

For FICI analysis, pH values were coded as 4.5 (1) and 5.5 (0), and bacterial species were coded as 1 for *Listeria* and 2 for *Salmonella*. In the MIC model, the bacteria variable was excluded since it was not significant.

## Data Availability

The original contributions presented in this study are included in the article/Supplementary Material. Further inquiries can be directed to the corresponding authors.

## Supplementary Materials

The following supporting information can be downloaded at: https://www.mdpi.com/article/10.3390/foods15162793/s1, Table S1: Concentrations (ppm) of ternary antimicrobial mixtures evaluated at pH 4.5 against *Listeria innocua* ATCC 51742 and *Salmonella* Typhimurium ATCC 14028, fractional inhibitory concentration index (FICI), and minimal inhibitory sum of concentrations (MIC). Table S2: Concentrations (ppm) of ternary antimicrobial mixtures evaluated at pH 5.5 against *Listeria innocua* ATCC 51742 and *Salmonella* Typhimurium ATCC 14028, fractional inhibitory concentration index (FICI), and minimal inhibitory sum of concentrations (MIC).
