# Supplementary material for: Mapping Antimicrobial Synergism in Sorbate-Based Ternary Preservative Systems Against Listeria innocua and Salmonella Typhimurium Using Multivariate Analysis
Source: Foods. 2026 Aug 10;15(16):2793. doi: 10.3390/foods15162793 (PMC13512305; doi:10.3390/foods15162793)
Supplement: Supplementary file 1 [file foods-15-02793-s001.zip › foods-4452502-supplementary.pdf]

Supplementary material

Table S1. Concentrations (ppm) of ternary antimicrobial mixtures evaluated at pH 4.5 against *Listeria innocua* and *Salmonella* Typhimurium, fractional inhibitory concentration index (FICI), and minimal inhibitory sum of concentrations (MIC)

Listeria innocua

Potassium sorbate	Thymol	Carvacrol	Eugenol	Citral	Vainillin	FICI	MIC
128	15	15				0.456	158
64	15		30			0.378	158
128	30		20			0.556	109
256	15		40			0.812	178
64	30		40			0.528	311
128	15		60			0.656	134
64	30		60			0.628	203
256	30			30		0.787	154
32	45			30		0.439	316
8	45			60		0.466	107
256	15			90		0.837	113
32	30			90		0.489	361
128	15			120		0.656	152
128	30				45	0.473	263
64	45				45	0.445	203
128	30				90	0.489	154
256	15				135	0.662	248
32	30				135	0.314	406
256	15				180	0.679	197
128		30	20			0.556	451
64		45	20			0.528	178
32		60	20			0.564	129
64		30	40			0.528	112
32		45	40			0.564	134
64		30	60			0.628	117
32		15	80			0.564	154
256		15		30		0.687	127
128		30		30		0.531	301
128		15		60		0.506	188
32		30		60		0.414	203
64		15		90		0.453	122
256		45			45	0.829	169

64		60			45	0.545	346
256		30			90	0.745	169
128		45			90	0.589	376
128		15			135	0.406	263
32		30			135	0.314	278
256			20	90		0.837	197
128			40	90		0.681	366
64			20	120		0.528	258
128			40		90	0.489	204
128			60		90	0.589	258
128			20		135	0.406	278
128			40		135	0.506	283
128			20		180	0.423	303
256				90	90	0.770	328
64				60	135	0.328	436
256				30	180	0.654	259

Salmonella Typhimurium

Potassium sorbate	Thymol	Carvacrol	Eugenol	Citral	Vainillin	FICI	MIC
128	15	15				0.456	158
16	15	30				0.252	61
256	15		20			0.692	291
32	30		20			0.344	82
32	15		40			0.324	87
128	15		60			0.596	203
256	30			30		0.812	316
32	45			30		0.464	107
16	45			60		0.532	121
128	15			90		0.656	233
16	30			90		0.532	136
128	15			120		0.756	263
128	30				45	0.606	203
64	45				45	0.578	154
128	30				90	0.756	248
256	15				135	1.002	406
8	30				135	0.666	173
128	15				180	0.956	323
256		15	20			0.692	291
64		30	20			0.408	114
32		45	20			0.444	97
8		60	20			0.496	88
256		15	40			0.772	311
32		30	40			0.424	102

32	45	40			0.524	117
256	15	60			0.852	331
32	30	60			0.504	122
32	15	80			0.484	127
256	15		30		0.712	301
64	30		30		0.428	124
32	45		30		0.464	107
256	15		60		0.812	331
32	30		60		0.464	122
128	15		90		0.656	233
256	45			45	0.962	346
128	60			45	0.806	233
256	45			90	1.012	391
128	15			135	0.806	278
32	30			135	0.714	197
256		20	90		0.892	366
128		40	90		0.716	258
256		80		45	0.982	381
256		40		90	0.972	386
64		60		90	0.668	214
256		20		135	1.002	411
128		40		135	0.866	303
128		20		180	0.936	328

Table S2. Concentrations (ppm) of ternary antimicrobial mixtures evaluated at pH 5.5 against *Listeria innocua* and *Salmonella* Typhimurium, fractional inhibitory concentration index (FICI), and minimal inhibitory sum of concentrations (MIC)

Listeria innocua

Potassium sorbate	Thymol	Carvacrol	Eugenol	Citral	Vainillin	FICI	MIC
16	60		50			0.527	126
16	75		50			0.602	141
128	60			60		0.613	248
8	60			120		0.513	188
16		50	50			0.560	116
8		60	50			0.613	118
8		80	50			0.747	138
64		20	100			0.640	184
128		40		180		0.780	348

256		50		160		1.027	466
8		60		160		0.680	228
8			150	60		0.713	218
16			150		160	0.680	326
128			75		240	0.593	443
8			100		240	0.493	348

Salmonella Typhimurium

Potassium sorbate	Thymol	Carvacrol	Eugenol	Citral	Vainillin	FICI	MIC
8	90	20				0.473	118
8	120	20				0.593	148
16	60		50			0.517	126
16	75		50			0.577	141
256	15		100			0.987	371
8	30		100			0.633	138
8	45		100			0.693	153
256	75				80	0.552	411
128	90				80	0.363	298
256		40	50			0.827	346
128		50	50			0.663	228
8		60	50			0.513	118
8		80		60		0.585	148
8		60			160	0.580	228
8			150	60		0.785	218
8			100		240	0.866	348
